# Reconciling Flux Experiments for Quantitative Modeling of Normal and Malignant Hematopoietic Stem/Progenitor Dynamics

**DOI:** 10.1016/j.stemcr.2021.02.020

**Published:** 2021-03-25

**Authors:** Munetomo Takahashi, Melania Barile, Richard H. Chapple, Yu-jung Tseng, Daisuke Nakada, Katrin Busch, Ann-Kathrin Fanti, Petter Säwén, David Bryder, Thomas Höfer, Berthold Göttgens

**Affiliations:** 1 Wellcome and MRC Cambridge Stem Cell Institute, University of Cambridge, Jeffrey Cheah Biomedical Centre, Cambridge Biomedical Campus, Cambridge CB2 0AW, UK; 2 Graduate School and Faculty of Medicine, The University of Tokyo, Tokyo 113-0033, Japan; 3 Department of Molecular & Human Genetics, Baylor College of Medicine, Houston, TX, USA; 4 Division of Cellular Immunology, German Cancer Research Center (DKFZ), Heidelberg, Germany; 5 Division for Molecular Hematology at Institute for Experimental Medical Sciences, Lund University, Lund, Sweden; 6 Department of Microbiology and Immunology at the Institute of Biomedicine, Göteborg, Sweden; 7 Division of Theoretical Systems Biology, German Cancer Research Center (DKFZ), Im Neuenheimer Feld 280, 69120 Heidelberg, Germany

**Keywords:** hematopoietic stem cells, mathematical modeling, fate mapping, reconcile different published data, cell dynamics, cell behaviour, kinetics

## Abstract

Hematopoiesis serves as a paradigm for how homeostasis is maintained within hierarchically organized cell populations. However, important questions remain as to the contribution of hematopoietic stem cells (HSCs) toward maintaining steady state hematopoiesis. A number of *in vivo* lineage labeling and propagation studies have given rise to contradictory interpretations, leaving key properties of stem cell function unresolved. Using processed flow cytometry data coupled with a biology-driven modeling approach, we show that *in vivo* flux experiments that come from different laboratories can all be reconciled into a single unifying model, even though they had previously been interpreted as being contradictory. We infer from comparative analysis that different transgenic models display distinct labeling efficiencies across a heterogeneous HSC pool, which we validate by marker gene expression associated with HSC function. Finally, we show how the unified model of HSC differentiation can be used to simulate clonal expansion in the early stages of leukemogenesis.

## Highlights

• A unifying mathematical model reconciles interpretations of three similar experiments
• Model quantifies HSC heterogeneity and hematopoietic self-renewal and differentiation
• Validation is performed via an independent test dataset and FACS analysis
• Applicability of the model is shown via simulations of perturbed scenarios

## Teaser

In this article, Takahashi, Barile, Göttgens, Höfer, and colleagues demonstrate that a unifying mathematical model can be used to reconcile opposing headline statements of hematopoietic stem cell contributions to hematopoiesis. Their model is validated by both an independent test dataset and FACS analysis. Thus, their work resolves a long-running dispute about the cell kinetics that underpin native hematopoiesis.

## Introduction

More than a century of sustained research efforts has established hematopoiesis as a paradigm for adult stem cell biology (Laurenti and Göttgens, 2018; Pappenheim, 1896). Sophisticated transplantation assays have pinpointed the key stem cell properties of self-renewal and multilineage differentiation capacity to a rare population of cells, present at a frequency of approximately 1 in 20,000 in mouse bone marrow (Seita and Weissman, 2010; Spangrude et al., 1988). Coupling transplantation with flow-cytometric cell sorting established an experimentally tractable differentiation hierarchy from hematopoietic stem cells (HSCs) via multipotent progenitors (MPPs) toward the individual blood lineages. Moreover, the initially defined HSC population was subsequently shown to be separable into long-term HSCs and short-term HSCs (ST-HSCs), of which only the former has true long-term (over 20 weeks) transplant reconstitution capacity, as well as the ability to maintain stem cell function through serial transplantation (Laurenti and Göttgens, 2018).

The high turnover of the blood system necessitates the constant production of large numbers of new blood cells. Pioneering transplant experiments following exposure to the chemotherapy agent 5-fluorouracil revealed that a pool of slowly cycling stem/progenitor cells exists upstream of faster cycling MPPs (Hodgson and Bradley, 1979). More recent experiments have established that those mouse HSCs with the most robust transplant ability tend to be the most quiescent HSCs (Oguro et al., 2013), with rates of cell division estimated to be less than once per 100 days (Wilson et al., 2008). Collectively, these studies raised the question as to how important HSCs are for native, unperturbed hematopoiesis, regardless of the undoubtedly pivotal role they play in transplant settings (Höfer and Rodewald, 2018). Transposon-based transgenic mouse models permit the labeling of individual stem/progenitor clones, and subsequently track how individual clones contribute to mature hematopoietic lineages over time. Using this approach, it was argued that long-lived progenitors, rather than classically defined HSCs, are the main drivers of unperturbed hematopoiesis (Sun et al., 2014). However, the technology could suffer from low sensitivity to detect clones within the HSC compartment, particularly those that divide rarely.

Several groups therefore embarked on label-propagation studies, where recombinase-mediated activation of fluorescent reporter genes creates a genetic label exclusively in HSCs (Busch et al., 2015), or in HSCs and, to a minor extent, intermediate progenitors (Chapple et al., 2018; Sawai et al., 2016; Säwén et al., 2018). The genetic label can be followed when HSCs enter differentiation to contribute to the various blood cell lineages. These four separate studies reported conflicting conclusions, ranging from minor to substantial contributions of HSCs to native hematopoiesis. Of note, one of the studies (Sawai et al., 2016) employed a strategy that entailed serial sampling of the same mouse, whereas the other three studies (Busch et al., 2015; Chapple et al., 2018; Säwén et al., 2018) utilized the same experimental design of using unique mice for each time point, thus facilitating direct comparisons.

Two of the four studies inferred the differentiation rates of HSCs and MPP cells by constructing mathematical models, built from the quantitative measurements of the speed at which the genetic label progresses down the hematopoietic hierarchy. Although the two studies inferred a similar order of magnitude for HSC output, around 1% (Busch et al., 2015) to 3% (Sawai et al., 2016) of HSCs per day giving rise to ST-HSCs, Sawai et al. (2016) found no self-renewing progenitors downstream of HSCs, calling for a major role for HSCs in sustaining hematopoiesis. Furthermore, some key assumptions for model construction were different (Pucella et al., 2020).

**Figure 1:**
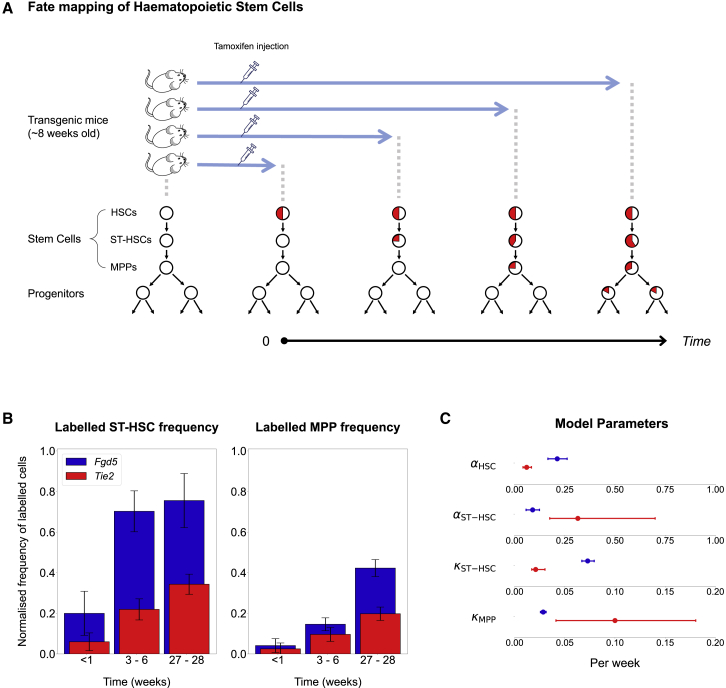
Published Label-Propagation Results with *Tie2* and *Fgd5* Cre Mouse Models Are Qualitatively Different (A) Outline of tamoxifen-inducible Cre recombination-based fate-mapping experiments to analyze stem cell kinetics. Healthy transgenic mice are injected with tamoxifen, which causes labeling of the HSC population. Circles represent stem cell populations with the proportion of labeled cells shown in red. Label propagates over time and appears sequentially in downstream populations. (B) Frequency of labeled cells in the ST-HSC and MPP compartments scaled to the labeling frequency in HSCs, following a uniform pipeline for flow cytometry data processing applied to data from Busch et al. and Säwén et al. Bars represent average and SEM at comparable time points (n = 104 or n = 48, respectively, for the three time points altogether, all independent experiments). (C) Model parameters of kinetic rates as inferred by the basic steady-state model developed for Busch et al. Best-fit value and the 95% profile likelihood confidence bounds are shown. Non-overlapping bounds highlight that this approach fails to find consistent parameters between the two studies. ⍺, differentiation rates; κ, measure of self-renewal, with smaller κ’s representing higher self-renewal in a compartment. Blue dots are computed parameters for *Fgd5* dataset, red for *Tie2* dataset.

In this study, we set out to resolve the different conclusions reported by the comparable lineage-tracing studies by using published data from the different groups. Direct comparison of labeled and unlabeled cell counts over time revealed clear qualitative and quantitative differences (see Results and Figures 1B and 1C), which we reasoned were due to the use of different transgenic Cre models. We therefore adopted a modeling approach that accounts for differences in label induction across a heterogeneous stem cell compartment (Barile et al., 2020). Importantly, this allowed us to define a single set of inferred kinetic properties that can explain training as well as validation datasets, thus demonstrating true predictive capability. As a result, we show how previous claims of contrasting degrees of HSC contribution to unperturbed hematopoiesis can be readily reconciled. Our study therefore provides a unified quantitative model for unperturbed hematopoiesis, which we furthermore exploit to interrogate the relationship between oncogene strength and target cell for transformation during the early stages of leukemogenesis.

## Results

### *Fgd5* and *Tie2* (Tek) Cre Knockin Elicits Qualitatively Different Label Propagations

Two recent lineage-tracing studies employed inducible Cre knockin alleles driven by *Fgd5* (Gazit et al., 2014; Säwén et al., 2018) and *Tie2* (Busch et al., 2015) regulatory elements to quantify label propagation across the hematopoietic hierarchy under unperturbed settings (Figure 1A). Both studies used flow cytometry to assess label propagation over an extended time course, Busch et al. (2015) inferring an HSC rate of differentiation into ST-HSCs of about 1% per day, whereas Säwén et al. (2018) suggested a more active HSC contribution, but without quantification. Given the central importance of this debate for the wider field of hematopoiesis, we set out to assess how these different conclusions may have arisen. We focused on the most immature populations in the hematopoietic hierarchy, namely LSK CD150^+^ CD48^−^ HSCs, LSK CD150^−^ CD48^−^ ST-HSCs, and LSK CD150^−^ CD48^+^ MPPs.

A direct comparison of the labeling frequency over time showed a faster accumulation of labeled cells in several downstream compartments (Figure 1B) for the *Fgd5* dataset. With the temporal dynamics of label propagation clearly different between the two datasets, we next asked whether the steady-state model of hematopoiesis developed for the *Tie2* dataset (Busch et al., 2015) could also explain the temporal dynamics observed in the *Fgd5* experiment (Säwén et al., 2018). The model describes stem and progenitor cell population dynamics, based on two parameters per population: the differentiation rate, i.e., the frequency at which progeny are produced from a progenitor, and the residence time, a measurement of a population's self-renewal degree. Deriving these parameters from the two different datasets resulted in substantially different values (Figure 1C). For example, the derived differentiation rate of HSCs for the *Fgd5* dataset was higher than for the *Tie2* dataset. Moreover, while a population downstream of HSCs with high self-renewal capability was inferred for both datasets, this comprised MPPs in Säwén et al. (2018) and ST-HSCs in Busch et al. (2015).

These analyses suggest that the underlying biology captured by the *Fgd5* versus the *Tie2* Cre model is distinct and that the steady-state model is of limited applicability. Moreover, the steady-state model did not provide a satisfactory fit to the *Fgd5* dataset (Figure S1A), whereas it did for the *Tie2* dataset, suggesting that a non-steady-state model (Barile et al., 2020) is required for a unified description of both datasets.

### Existing Models Do Not Address Key Aspects of Stem Cell Behavior

Computational modeling has played a pivotal role in efforts to infer stem cell kinetics and has uncovered properties of stem cells that could otherwise not have been deduced from static analysis of the data alone (Buchholz et al., 2013; Foudi et al., 2009; Mackey, 2001; van der Wath et al., 2009). However, our analysis in the previous section showed that the approaches taken previously to model HSC label propagation lack the flexibility to explain more than a single experimental dataset. The mathematical model in Busch et al. (2015) assumes that the flow-cytometric HSC population is homogeneous and characterized by uniform cell kinetic rates, while a more recent extension of this model accounts for HSC and ST-HSC population heterogeneity (Barile et al., 2020). Whereas the former model cannot account for the increase over a time span of 2 years in the frequency of labeled HSCs, observed in all lineage-tracing datasets (data not shown), the latter model does. Given that Cre induction occurred only at the beginning of the time course, the most likely explanation for a subsequent increase in the proportion of labeled cells in the flow-cytometric HSC population is that this population is heterogeneous, where a subpopulation displays higher labeling frequency right from the start, which subsequently propagates to the rest of the population. Moreover, the homogeneity assumption of the simple steady-state model prevents the model from fitting the plateau of ST-HSC normalized label frequency. With a homogeneous HSC population, this frequency is expected to converge to 1, but as observed in the *Fgd5* dataset (Figure S1A), the frequency tails off at around 0.7.

Another simplification of the published models is the assumption of a steady state in Busch et al. (2015) and Sawai et al. (2016) with constant compartment sizes and rates during a mouse’s life. Barile et al. (2020) and Bernitz et al. (2016) show that several populations, including the HSC population, change substantially with aging along extended time courses (see Figure S1B). Finally, the assumption of time-independent rates does not consider the decrease in stem cell output upon aging (as observed by Säwén et al., 2018, and Barile et al., 2020). As the overarching purpose of modeling is the derivation of biologically meaningful conclusions that are statistically supported by all the available experimental data, we asked whether the *Tie2* and *Fgd5* datasets could be reconciled by an extended non-steady-state model with HSC heterogeneity.

### HSC Population Heterogeneity Suffices to Explain Quantitative Differences When Using *Fgd5* and *Tie2* Cre Drivers

**Figure 2:**
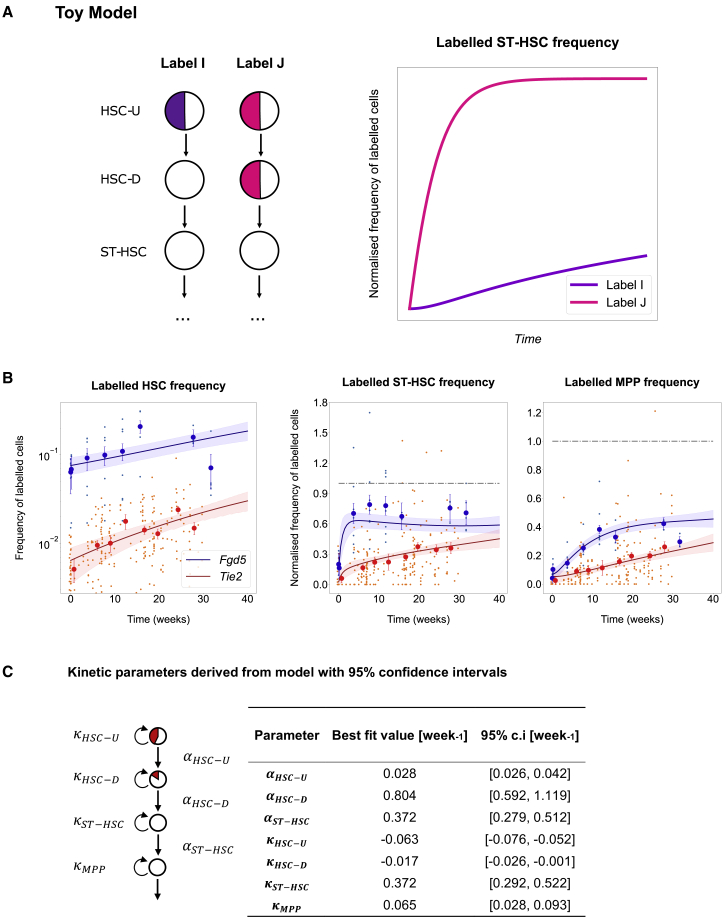
Different Initial Labeling of HSC Subcompartments Explains the Qualitative Differences among Datasets (A) Concept illustrated by a toy model. Left: a differentiating cascade with a stem cell population of HSCs producing progeny ST-HSCs. HSCs are heterogeneous, but treated as homogeneous due to the lack of markers to distinguish the subcompartments. HSC-U, upstream; HSC-D, downstream. Two hypothetical Cres, I and J, label the upstream and downstream compartments with different proportions. Right: the simulated accumulation of labeled cells in ST-HSC, normalized to the labeling frequency of HSCs considered as homogeneous, is qualitatively different for labels I and J, although the underlying population kinetics are the same. (B) Model best fit (solid line) and 95% prediction profile likelihood confidence bounds on the model (shaded area) plotted against the experimental data (big dots representing average and SEM of datasets, small dots the mice from independent experiments, n = 242 or n = 48, respectively, for *Tie2* and *Fgd5* datasets for all three plots). The variance was pooled for all data points to account for the fact that later time points have different numbers of samples, reflected in the larger confidence bounds on parameters and model. Kinetics appear qualitatively different between the two datasets due to the different initial labeling frequencies. Data are an average of all the available data, measured at common time points. Labeled frequencies of ST-HSC and MPP populations have been normalized to the labeled frequency of HSCs. (C) Best-fit model parameters trained on the two datasets, shown alongside 95% prediction profile likelihood confidence bounds. The differentiation rate of HSC-D is high, suggesting a major contribution to hematopoiesis, although the confidence bounds are also large.

Heterogeneity within flow-cytometric HSC populations has long been recognized and explored experimentally by several groups (Oguro et al., 2013; Sawai et al., 2016; Wilson et al., 2008). HSC heterogeneity has also been considered in tissue models (Roeder and Loeffler, 2002), but so far has not been used to integrate different label-propagation experiments. We therefore investigated whether quantitative differences between the *Fgd5* and the *Tie2* label-propagation datasets could be explained by a heterogeneous HSC population, where the subpopulations are labeled with different probabilities by the *Fgd5* versus the *Tie2* Cre. Figure 2A illustrates this concept with a toy model. Let us assume that HSCs are split into two subcompartments (upstream [HSC-U] and downstream [HSC-D]). If two different Cre drivers (label I and label J) were to label the upstream and downstream subcompartments 50% and 0% versus 50% and 50%, respectively, then the time-dependent accumulation of labeled cells in population ST-HSC would be qualitatively different, even though the kinetic parameters of all the populations are the same.

We thus adopted the idea of a heterogeneous HSC population and dropped the steady-state assumption. To satisfy the constant rate assumption, we narrowed the observation window to 40 weeks, thus excluding the major aging-related alteration in population abundance within the extended time-course datasets.

This new model was fit to both the *Fgd5* and the *Tie2* datasets (both the frequency and the population size data) simultaneously to infer common underlying kinetics, but permitting different initial labeling frequencies for each population. The model fit provided a good description of the input data, with most points lying within the prediction profile likelihood (Kreutz et al., 2012) confidence bounds (Figure 2B) for both label frequency and compartment population size. Modeled population sizes over time revealed substantial confidence bounds for the inferred most immature HSC population, which was predicted to expand (Figure S2). Of note, all estimated parameters have at least one identifiable bound, so the model is not overfitted (Murray 2002; Raue et al., 2009). Even though the datasets appear to infer different biological properties at first glance, both datasets are consistent with a single set of stem cell kinetics as long as differences in the initial Cre-mediated labeling are considered.

**Figure 3:**
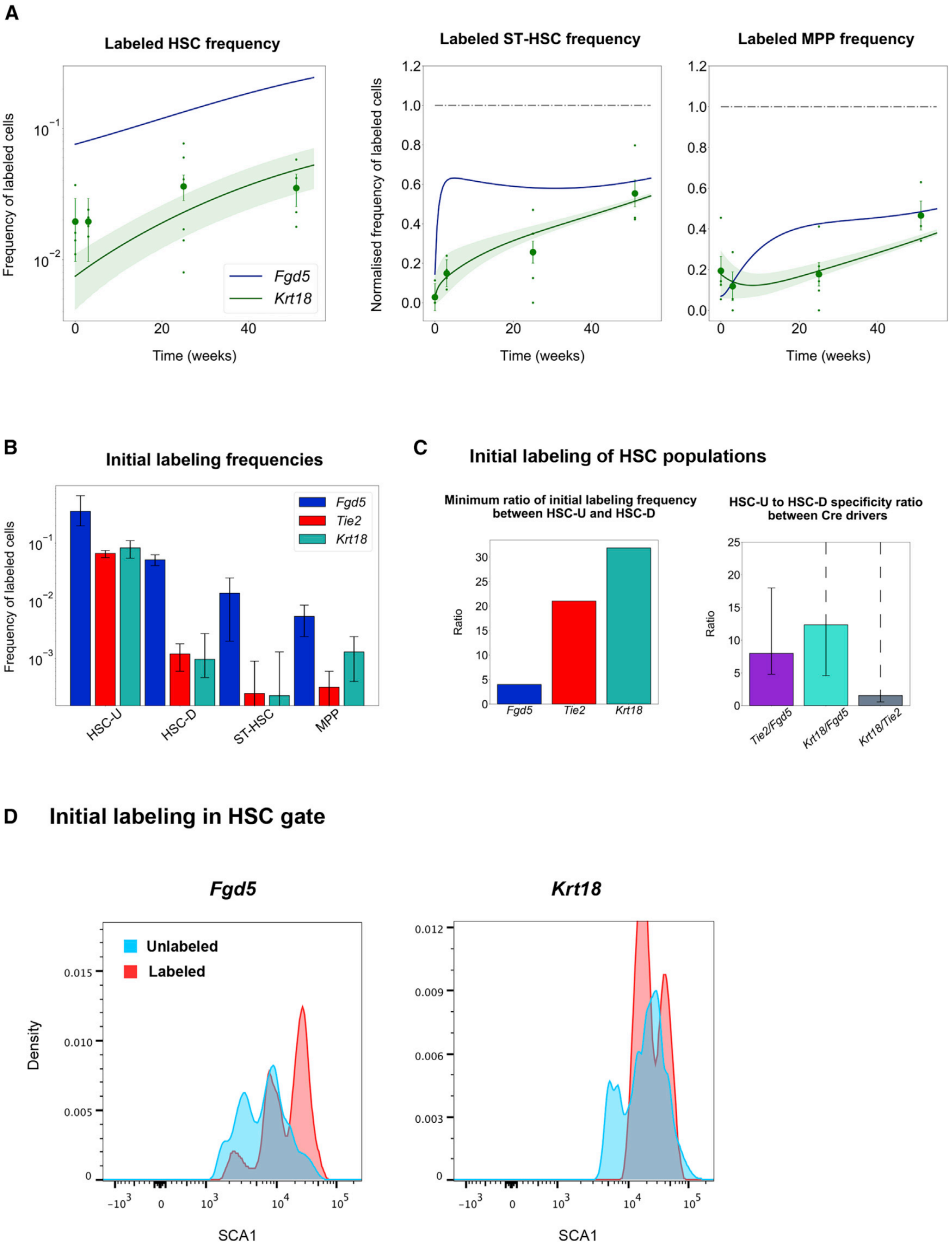
Investigation of Model Parameters Gives Insights into Cre Labeling Marker Bias and Stem Cell Kinetics (A) Predicting an independent experiment from the learned kinetics. Upon changing only the initial labeling frequency in the HSC-U and HSC-D populations, an independent experiment can be predicted with the best-fit kinetics learned from the previous two datasets. Blue line, best fit for the *Fgd5* dataset. Green, best fit (solid line) and 95% profile likelihood confidence bounds (shading) on the model for the *Krt18* datasets. Big dots represent average and SEM of dataset, small dots the mice from independent experiments, n = 54 for all three plots. (B) Model-predicted initial labeling frequencies for each stem cell population. The three Cre drivers are predicted to label each population by different amounts, leading to an observable difference in the temporal dynamics. Error bars show confidence intervals for parameters. (C) Model-predicted initial labeling frequency ratios of HSC-U to HSC-D. Left side shows the minimum value for the ratio of initial HSC-U labeling frequency to initial HSC-D labeling frequency for each Cre driver. Right side shows the ratio of these ratios between the different Cre drivers. It can be interpreted as the specificity of a Cre driver to HSC-U over HSC-D. Error bars show confidence intervals for parameters with dotted line indicating unbounded parameter. (D) SCA1 expression-density plots for HSCs in *Fgd5* and *Krt18* fate-mapping experiments. Plots show a comparison of the distribution between labeled and unlabeled HSCs for each reporter gene at the first measured time point after induction (7 days).

In particular, the key difference inferred by the model relates to different labeling of the HSC subcompartments with the *Tie2* and *Fgd5* Cre knockin mice (Figure 3B).

The inferred ratio of initial labeling frequency between HSC-U and HSC-D (Figure 3C) is at least 4:1 for *Fgd5* and 21:1 for *Tie2*. Consequently, *Tie2* Cre-mediated labeling is inferred to be at least 4.8 times more specific to HSC-U than the *Fgd5* Cre-induced label. Intuitively, this suggests that with *Fgd5* Cre, labeled cells from HSC populations as defined by flow cytometry will progress faster to the ST-HSC population because of the relatively higher HSC-D labeling frequency, since HSC-D is “closer” to ST-HSC than HSC-U.

Taken together, our results suggest that different initial labeling frequencies of a heterogeneous HSC population are sufficient to explain the observed differences between *Fgd5* and *Tie2* Cre-induced label-propagation studies. Importantly therefore, both studies are consistent with a common and unified set of underlying stem cell kinetics.

### The Unified Stem Cell Kinetics Inferred for the *Tie2* and *Fgd5* Datasets Explain a Third Independent Fate-Mapping Experiment

The initial requirement for any model designed for experimental data is that it accurately captures a set of experimental observations. However, a stringent test of the predictive power of a computational model is its application to an independent experiment that did not form part of the training data. We therefore used the same flow cytometry processing pipeline to analyze a third independent lineage-tracing/label-propagation study from Chapple et al. (2018). This study used a Cre transgene exploiting gene regulatory sequences of the *Krt18* gene locus to drive expression of an inducible Cre transgene in the HSC compartment. Importantly, this allowed us to validate the idea that different initial labeling frequencies of stem cells, particularly of a heterogeneous HSC population, are sufficient to unify experimental observations from different studies, with all stem/progenitor cell kinetics remaining fixed (and thus the same) across all three studies.

Stem cell kinetics from the joint analysis of the *Fgd5* and *Tie2* datasets were fixed, and only the initial labeled frequencies were trained on the new *Krt18* data points (Figure 3A). This modeling approach provided a good fit for the *Krt18* dataset, thus not only demonstrating the predictive power of our model, but also emphasizing the notion that a single set of stem/progenitor cell kinetics is compatible with multiple label-propagation studies, which at first glance appeared to present highly contradictory results. Indeed, Chapple et al. (2018) had concluded, although without quantification, that the HSC population is active, in line with Säwén et al. (2018), but in contrast to Busch et al. (2015). Our analysis suggests that there is no need to assume any one of these studies to be incorrect. Instead, the supposed discrepancies can be reconciled by a revised interpretation of the properties of the data, which importantly is based on a single set of assumptions about the underlying biological properties of the stem cells.

### The Unified Model Predicts Inactive and Active HSC Subpopulations and Sustained Self-Renewal Downstream of HSCs

Our new approach for studying unperturbed hematopoiesis faithfully captures experimental label-propagation experiments from three different groups using three different Cre drivers. We were therefore interested to explore further the model parameters (Figure 2C), as they should have a significantly higher chance of capturing the true biology of unperturbed hematopoiesis than previous modeling efforts that, as we show here, cannot explain more than a single dataset. The model predicted an inactive near-quiescent upstream HSC-U population with around 1 in 50 HSC-U cells differentiating per week, and an active HSC-D population with each HSC-D cell differentiating every week, thus suggesting that at least a subset of HSCs contributes frequently to normal hematopoiesis, as emerged qualitatively from the Fgd5 dataset. On the other end, we identified MPPs as an almost self-renewing population (high residence time, low κ value), consistent with a key role for progenitors downstream of HSCs in supplying fresh blood cells in steady-state hematopoiesis as well as in situations where stem cell input may be lacking (Schoedel et al., 2016; Sheikh et al., 2016). Of note, while the model gives a clear indication of self-renewal downstream of HSCs, which subpopulations are identified as self-renewing may depend on the potential heterogeneity of the ST-HSC and MPP compartments.

Our model therefore reconciles the various headline statements from studies previously regarded as contradictory. Cells within the HSC pool readily contribute to hematopoiesis, but the model also shows that non-stem progenitor populations can have prominent roles in sustaining hematopoiesis. Overall, the parameters suggest that a subcompartment of the HSC population is indeed producing differentiated cells frequently, although downstream progenitors have a sufficient degree of self-renewal to cope with prolonged stem cell failure.

### Inferred HSC Labeling Patterns Agree with SCA1 Surface Marker Expression

As outlined above, the new model has a constant set of kinetic parameters but assumes that (1) the HSC population is composed of two hierarchically connected subpopulations, and (2) the different Cre drivers label cells in the two subcompartments with different proportions. The inferred ratios of labeling frequencies for HSC-U and HSC-D for the three Cre drivers are at least as follows: 4:1 for *Fgd5*, 21:1 for *Tie2*, and 30:1 for *Krt18* (Figures 3B and 3C). To test these inferred proportions, we investigated the fluorescence-activated cell sorting intensities of the SCA1 surface marker for labeled and unlabeled HSCs (Figures 3D and S3). Previous studies have shown a link between SCA1 expression levels and the reconstitution potential of the HSC population after transplantation (Wilson et al., 2015), with SCA1^high^ cells providing more robust and durable long-term reconstitution than SCA1^lo^ cells. SCA1^high^ cells are thus thought to account for the long-term reservoir of dormant HSCs (Sawai et al., 2016; Wilson et al., 2008).

Fortuitously, Chapple et al. (2018) reported lineage propagation data not just for the *Krt18* Cre, but also for the same *Fgd5* Cre (as used by Säwén et al., 2018). This allowed us to perform robust quantitative comparisons of flow cytometry intensities, which would be rather challenging to perform on datasets generated by different groups using different machines. This comparative analysis demonstrated that HSCs labeled with *Krt18* have higher SCA1 levels compared with unlabeled HSCs, while SCA1 expression of HSCs labeled with *Fgd5* spills over to the lower-intensity peaks, and thus overlaps more broadly with the unlabeled HSCs. Given the known association of the most robust and durable transplantable HSCs with high levels of SCA1, this result is consistent with our inferred differential labeling efficiencies for the *Krt18* and *Fgd5* Cre drivers, since the modeling had inferred that *Krt18* Cre would show higher labeling specificity for the most upstream HSC subpopulation. This analysis therefore provided experimental validation and corroborated our approach of inferring values for differential HSC labeling patterns across a heterogeneous HSC compartment. Moreover, these findings highlight the notion that opposing conclusions drawn from the various label-propagation studies are indeed a likely consequence of different biological properties of the various Cre drivers.

### Modeling Leukemia Development Kinetics Based on Cell of Origin

A model describing the kinetics of stem cells can be used to give insights into disorders that arise in stem or progenitor cells. Since our combined model appears robust in capturing biological features of normal hematopoiesis, we were interested to see whether the model could also be used to simulate the early steps of malignant transformation toward leukemia. More specifically, we wanted to interrogate the potential dependence of leukemic progression on the population where the mutation first arises. Leukemogenesis commonly develops as a stepwise process, where preleukemic clones show normal differentiation into all the mature lineages, yet have a so-called clonal advantage, whereby the clone expands as a fraction of the entire system over time. Within our model, this process can be investigated by tuning the parameters inferred for normal hematopoiesis to simulate the emergence of a malignant cell, and then simulating how this “clone” behaves over time.

**Figure 4:**
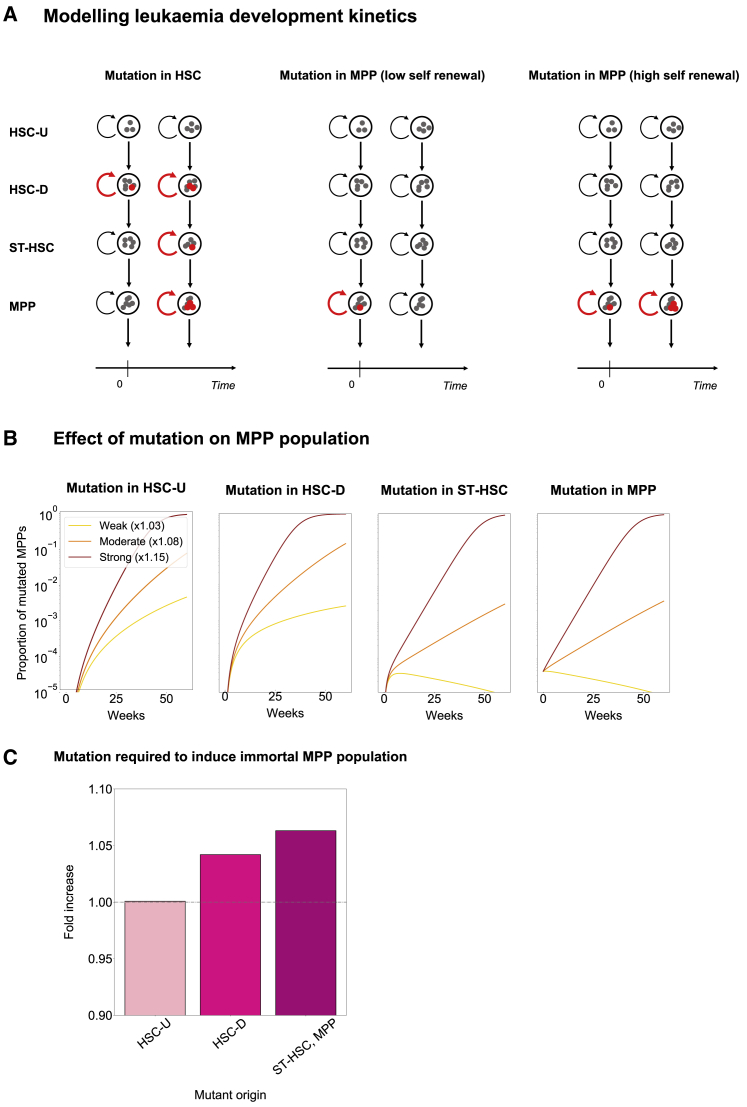
Modeling Cell of Origin Reveals Distinct Leukemogenesis Dynamics (A) Model to investigate propagation of single cell mutation from each stem cell population dependent on the factor by which cell proliferation increases. A single cell mutation in one population propagates to progeny populations over time. Whether the mutation population develops is dependent on population of origin and degree of increase in self-renewal (i.e., strength of mutation). (B) Simulation of leukemogenesis dynamics over 60 weeks compared by mutant cell origin. Plots show proportion of MPP cells that are mutants (have higher proliferation rates). A strong mutation in any stem cell population causes an overshoot of mutants in the MPP population (red). However, the effect of a weak/moderate mutation is highly dependent on cell of origin (orange, yellow). (C) Simulation of leukemogenesis dynamics to induce immortal mutant MPP population. Immortal population can be induced by any stem cell population, but the strength of mutation required is highly dependent on mutant origin. Bars show the fold increase of the proliferation rate in each population with respect to its normal haematopoisis value.

Based on the population kinetics from our new combined model, we first investigated how a mutant stem cell with enhanced proliferation would cause an accumulation of the progeny of this mutant clone into the MPP compartment (Figure 4A). Given the active debate on whether ST-HSCs and/or MPPs can serve as cells of origin for leukemia development, we also simulated the emergence of individual mutant clones in these cell populations. For modeling purposes, we assumed that the initial mutation would endow the target cell and all of its daughter cells with an increased proliferative potential, and thus tested proliferation rate increases by factors of ×1.03 (a weak mutation), ×1.08 (a moderate mutation), and ×1.15 (a strong mutation). Following the model for a 60 week time course showed that leukemic clone establishment was dependent on both the cell of origin and the strength of the mutation (Figure 4B). “Weak” oncogenic mutations in the upstream cell populations, namely HSC-D and HSC-U, result in the establishment of the leukemic clone, but “weak” oncogenic mutations originating in the downstream cell populations are diluted out and are insufficient to promote preleukemic development. By contrast, “strong” mutations are sufficient to cause clonal expansion, regardless of the cell of origin.

We next identified the threshold of mutation strength required to generate an expanding clone starting from the four cell types contained within our model. We found that a mutation in all cell populations can induce an immortal mutant clone population in the MPP compartment (Figure 4C), but again, the ease with which this is achieved is dependent on the cell population. For HSC-U, any increase in proliferation is enough, for HSC-D an increase of ×1.04 is required, and for ST-HSC and MPPs, this value is ×1.06. The relationship between mutation strength and cell of origin extended all the way to the two HSC subpopulations. In terms of potential mechanisms, these findings suggest that a very weak oncogenic mutation in the HSC population is sufficient to cause mutant cells flowing into the MPP population to balance the loss of mutant cells due to further differentiation. However, a stronger oncogenic mutation is required to create a similar immortal mutant MPP population if the initiating mutation arises within the MPP themselves. From a clinical perspective, our results serve as a potential explanation of how leukemic clones with identical mutations may exhibit different levels of “fitness” depending on their cell of origin, and thus may respond differently to treatment.

## Discussion

Recent advances in lineage tracing and genomic technologies are ushering in a new era of stem cell biology, with the goal of defining stem cell/progeny relationships at both the cellular and the molecular level. It is widely expected that the resulting deep understanding of stem cell differentiation trajectories will provide a blueprint to better understand perturbations that drive stem cell diseases, as well as informing the development of directed differentiation protocols to produce the desired cell types for either drug discovery or cellular therapy. However, vastly different conclusions have been drawn from the initial label-propagation studies performed using HSC-specific (Busch et al., 2015) and HSC- and ST-HSC/MPP-specific (Chapple et al., 2018; Sawai et al., 2016; Säwén et al., 2018) Cre driver mouse models. Our study presents an approach that reconciles the different studies, provides insights into what caused the divergent interpretations, and demonstrates how a refined computational model anchored in biology can be utilized to quantify aberrations in cellular function that may drive the early stages of leukemogenesis.

We would argue that the problem that we have addressed here is broadly relevant to all areas of biology where modeling is employed to develop a useful abstraction of the underlying processes. For our comparisons between datasets, we took account of three levels, namely a qualitative description of the data, quantitative interpretation, and the inferred biological mechanisms. Comparisons based on qualitative or quantitative descriptions alone can fail to identify fundamental similarities in the underlying biological mechanism, particularly because models are only accurate descriptions of our thinking (Gunawardena, 2014). Similarly, the same biological mechanism can manifest itself in different qualitative and quantitative descriptions. Fitting both datasets at the same time allowed us to counteract these problems and to focus on the underlying biological mechanism. Just as we were able to reconcile the different studies on HSC activity, we believe that this approach may be useful to develop a consensus view in other areas of biomedical research where computational modeling applied to individual datasets has produced divergent conclusions.

The delineation of the initial versions of the hematopoietic differentiation tree represents one of the most significant accomplishments of stem cell research in the last two decades of the 20th century (Akashi et al., 2000; Kondo et al., 1997). Subsequent refinements have split many of the original populations into defined subpopulations, such as MPP1-4 for the MPPs (Adolfsson et al., 2005; Cabezas-Wallscheid et al., 2014; Pietras et al., 2015; Wilson et al., 2008). Functional assays have also revealed heterogeneity within the HSC population, for example, by identifying HSCs with particularly potent and durable reconstitution activity based on high SCA1 or medium KIT expression (Grinenko et al., 2014; Wilson et al., 2015). We reasoned that Cre drivers under the control of different regulatory sequences would differ in their relative labeling of HSCs across this spectrum of heterogeneous function, and that in turn such differential labeling could explain differences in the observed stem cell kinetics. Importantly, we validated differential labeling of SCA1-high HSCs by two of the transgenic cassettes, thus providing experimental evidence for our modeling approach, which was able to reconcile different studies based on differential labeling of a heterogeneous HSC compartment. The flux parameters for our unifying model predicted a near-quiescent HSC-U population and an active HSC-D population. There is both prior experimental and theoretical evidence supporting our approach (Bernitz et al., 2016; Glauche et al., 2007; Morita et al., 2010; van der Wath et al., 2009; Wilson et al., 2008). HSCs have commonly been divided into two populations based on either reconstituting capacity (Morita et al., 2010) or cell-cycle status (van der Wath et al., 2009) or a combination of the two (Oguro et al., 2013).

Our model is in general agreement with the size and characteristics of the subcompartment structure proposed by previous studies. In particular, it suggests that the upstream HSC population is at least five times smaller than the downstream HSC population, in agreement with the idea of a hematopoietic hierarchy where the relative size of progenitor populations tends to increase as one progresses down the hierarchy (Busch et al., 2015; Sawai et al., 2016). Attempts to define heterogeneous subcompartments with lineage tracing have already been made (Barile et al., 2020). However, with the data currently available, the upstream HSC population still shows an increase in labeled cells over time, indicating that there is further population heterogeneity that is not yet resolved with commonly used markers. As a result, there is no consensus on whether the HSC population should be split into two or three, or, in the new era of single cell biology, whether we should even think of discrete subcompartments any longer. Nevertheless, our model shows that current experimental data can be well explained by considering two HSC subcompartments, whereby a minimal topology model that connects all populations can explain the data while being constrained using bounded parameters. Alternative topologies could still fit the model, but would either have more parameters or substantially deviate from the classical linear model of hematopoiesis. Although we cannot rule out these possibilities, we limited ourselves to the simpler linear case. Furthermore, a revised modeling approach will be needed to capture the behavior of aging HSPCs, given that there are likely to be changes in the underlying molecular processes that occur during aging, but are not accommodated in our current model. Novel experimental and computational approaches will be needed to ultimately define HSC stem cell kinetics at single-cell resolution.

Research over the past decade has revealed that hematopoietic malignancies are commonly preceded by prolonged periods of clonal hematopoiesis, where progeny from a single stem/progenitor cell make up a significant proportion of the entire blood system. Called either CHIP or ARCH, it is now widely recognized that clonal hematopoiesis is more the norm rather than the exception in aged individuals (Busque et al., 2018). Clonal expansion is also a hallmark of full-blown malignancies, where the relationship between “oncogene strength” and cell type of origin has already been explored experimentally. Specifically, it was shown that “stronger” oncogenes could transform more downstream progenitors, whereas “weaker” oncogenes needed to be introduced into the top tiers to cause malignant transformation (as reviewed by Horton and Huntly, 2012). Moreover, the JAK2V617F mutation associated with clonal hematopoiesis was shown to confer long-term repopulation ability onto downstream progenitor cells in a mouse model of myeloproliferative neoplasms (Lundberg et al., 2014). Our computational model allowed us to capture the dependence of oncogenic mutation strength on the target cell for mutation, commonly referred to in leukemia research as the “cell of origin.” Weak mutations are washed out of the system if they occur in MPPs but cause sustained clonal expansion if they occur in HSCs. Moreover, the relative increase in self-renewal activity required to create an immortal MPP is small (1.06-fold increase), suggesting that (1) MPPs may represent the cell of origin for many leukemias due to their larger pool size compared with HSCs, and (2) many experimental techniques currently used will struggle to pick up such small changes in self-renewal activity.

Perhaps it is not a surprise, therefore, that sampling across the long time spans of human aging represents the most robust ways to characterize clonal hematopoiesis so far. However, studies relying on inferring clonal events retrospectively from analysis of human patient samples do not represent an experimentally tractable system. This in turn suggests a real need for computational modeling approaches, based on abstractions of the hematopoietic system that agree with experimental data across a range of different laboratories. We hope that the work presented here will stimulate further examination of both normal and perturbed hematopoiesis.

## Experimental procedures

### Steady-State Model Equations for Normal Hematopoiesis

The same model as used in Busch et al. (2015) was applied to the data obtained by Säwén et al. (2018). The model consisted of solving three ordinary differential equations using standard minimization of the sum of weighted square residuals. Best-fit parameters with 95% profile likelihood confidence bounds and the best-fit model with 95% prediction profile likelihood confidence bounds were inferred. Either standard error of the means or pooled variance was considered to weigh the squared residuals, but neither produced a good model fit (Figure S1A).

### Non-Steady-State Model Equations for Normal Hematopoiesis

The non-steady-state model used to fit both *Tie2* and *Fgd5* datasets was adapted from Barile et al. (2020). As in Busch et al. (2015), the expected number $n_{i}\left(t\right)$ of cells in a population i in a linear pathway obeys the following system of ordinary differential equations:

(Equation 1) $$\left\{ \begin{array}{c} \frac{dn_{1}\left(t\right)}{dt}=\;-\;\left(\alpha_{1\;}-\;\beta_{1}\right)\;n_{1}\left(t\right)\;\\ \frac{dn_{i>1}\left(t\right)}{dt}=\alpha_{i-1\;}n_{i-1}\left(t\right)-\;\left(\alpha_{i}-\beta_{i}\right)\;n_{i}\left(t\right) \end{array}\right..$$

The parameters represent the cells’ fates, where for each population, i represents the flux downstream and $\beta_{i}$ is defined as the net proliferation rate, i.e., the difference of cell proliferation $\lambda_{i}$ and death $\delta_{i}$:

(Equation 2) $$\beta_{i}=\;\lambda_{i}-\delta_{i}\;.$$

These kinetic parameters were assumed shared for both datasets and constant for the whole time frame. To understand the kinetics more intuitively, the cell inverse residence time $\kappa_{i}$ (inversely proportional to the amount of time required for a population to reduce to one-half of its initial size if the input is switched off) was defined as:

(Equation 3) $$\kappa_{i}=\alpha_{i}-\beta_{i}.$$

We assumed the labeled cells followed the same physiological behavior as the unlabeled cells. Thus, the expected number of labeled cells $l_{A,i}\left(t\right)$ for a reporter gene A in a given population i obeys equations analogous to Equation 1:

(Equation 4) $$\left\{ \begin{array}{c} \frac{dl_{A,1}\left(t\right)}{dt}=\;-\;\kappa_{1}\;l_{A,1}\left(t\right)\;\\ \frac{dl_{A,i>1}\left(t\right)}{dt}=\alpha_{i-1\;}l_{A,\;i-1}\left(t\right)-\;\kappa_{i}\;l_{A,i}\left(t\right) \end{array}\right..$$

To reduce measurement noise, instead of the number of labeled cells, the frequency $f_{A,i}\left(t\right)=\frac{l_{A,i}\left(t\right)}{n_{i}\left(t\right)}$ of labeled cells was used with the population size to parametrize the model:

(Equation 5) $$\left\{ \begin{array}{c} \frac{df_{A,\;1}\left(t\right)}{dt}=\;0\\ \frac{df_{A,i}\left(t\right)}{dt}=\alpha_{i-1\;}\frac{n_{i-1}\left(t\right)}{n_{i}\left(t\right)}\;\left(f_{A,\;i-1}\left(t\right)-f_{A,i}\left(t\right)\right) \end{array}\right..$$

We defined the populations in upstream-to-downstream order as HSC-U, HSC-D, ST-HSC, and MPP.

The initial conditions for Equation 1 (i.e., $n_{i}\left(0\right)$) and the initial labeling frequency for each population and each reporter gene (dataset) were additional parameters (i.e., $f_{A,i}\left(0\right)$).

### Data

The experimental data were obtained from Busch et al. (2015) (over 100 mice), Säwén et al. (2018) (over 50 mice), and Chapple et al. (2018) (18 mice). The original *Tie2* dataset was augmented by further experimental data measured post-publication and published in Barile et al. (2020) to improve precision. The time frames of the three experiments were standardized so that they could be compared directly. The measurement at time 0 was adjusted to be the frequencies observed 2 days after tamoxifen was assumed to have taken effect. For the *Krt18* dataset, owing to limited data points, time 0 was adjusted to be the frequencies observed 1 day after tamoxifen was assumed to have taken effect.

To limit noisy variation from the *Tie2* dataset, the data were pooled to nearby time points as follows: 0–20 days (n = 41), 27–50 days (n = 61), 55–69 days (n = 21), 78–95 days (n = 8), 104–129 days (n = 39), 130–153 days (n = 34), 160–179 days (n = 21), and 188–208 days (n = 17). The number of pooled categories was chosen to be equal to the number of separate time points in the *Fgd5* dataset (n = 8). To obtain a consistent estimate of the measurement error, the variances were pooled for all the data points for all the datasets to calculate the standard error of the mean.

Finally, for the non-steady-state model, the measurements of compartment population size from Busch et al. (2015) and Säwén et al. (2018) were combined, and the data were pooled into eight categories (Figure S1B).

### Model Fit

See the Supplemental Experimental Procedures for details about how fit was performed.

### Leukemogenesis Dynamics

Using the model parameters (α,κ,n(0)) derived from Equation 5, the number of mutant cells $m_{i}\left(t\right)$ in population i was assumed to obey equations analogous to Equations 1 and 2 such that:

(Equation 8) $$\left\{ \begin{array}{c} \frac{dm_{1}\left(t\right)}{dt}=\;-\;\kappa_{1}\;m_{1}\left(t\right)\;\\ \frac{dm_{i>1}\left(t\right)}{dt}=\alpha_{i-1\;}m_{i-1}\left(t\right)-\;\kappa_{i}\;m_{i}\left(t\right) \end{array}\right..$$

A mutation was modeled as a factor increase in proliferation rate, such that a mutation of ×1.03 would increase the cell proliferation rate $\lambda_{i}$ to $1.03\lambda_{i}$ for the mutated population and its downstream progenitors. Differing strengths of proliferation rate increases were applied to each population separately using the estimation of death rates from Barile et al. (2020) and the $\alpha_{\text{MPP}}$ estimate from Busch et al. (2015).

The proportion of mutated MPPs p(t)obtained from a mutation in population s was determined by setting the initial value of $m_{s}\left(0\right)$ as:

(Equation 9) $$m_{i}\left(0\right)=\;\left\{ \begin{array}{cc} 0 & i\neq s\\ 1 & i=s \end{array}\right.,$$

and obtaining the ratio:

(Equation 10) $$p\left(t\right)=\;\frac{m_{4}\left(t\right)}{n_{4}\left(t\right)}.$$

Last, the mutation required to induce an immortal MPP population was calculated computationally by altering the proliferation rate increase until $\lim_{t\to \infty }p\left(t\right)=constant$.

## Author contributions

B.G. and M.B. conceived and designed the study. M.B. and T.H. conceived the mathematical model. M.T. wrote all codes and performed all calculations. M.T., M.B., and B.G. wrote the paper. R.H.C., Y.-J.T., K.B., A.-K.F., and P.S. measured the data. D.N. and D.B. conceived the fate-mapping experiments and contributed to writing the manuscript. T.H. contributed to writing the manuscript. All authors have read and approved the manuscript.

## Conflicts of interest

The authors declare no competing interests.

## Supplemental information

Document S1. Supplemental Experimental Procedures and Figures S1–S3

Document S2. Article plus Supplemental information
