# Supplementary material for: Reconciling Flux Experiments for Quantitative Modeling of Normal and Malignant Hematopoietic Stem/Progenitor Dynamics
Source: Stem Cell Reports. 2021 Mar 25;16(4):741–53. doi: 10.1016/j.stemcr.2021.02.020 (PMC8072066; doi:10.1016/j.stemcr.2021.02.020)
Supplement: Document S1. Supplemental Experimental Procedures and Figures S1–S3 [file mmc1.pdf]

Stem Cell Reports, Volume *16*

Supplemental Information

Reconciling Flux Experiments for Quantitative Modeling of Normal and Malignant Hematopoietic Stem/Progenitor Dynamics

Munetomo Takahashi, Melania Barile, Richard H. Chapple, Yu-jung Tseng, Daisuke Nakada, Katrin Busch, Ann-Kathrin Fanti, Petter Säwén, David Bryder, Thomas Höfer, and Berthold Göttgens

Supplemental Figure 1

A

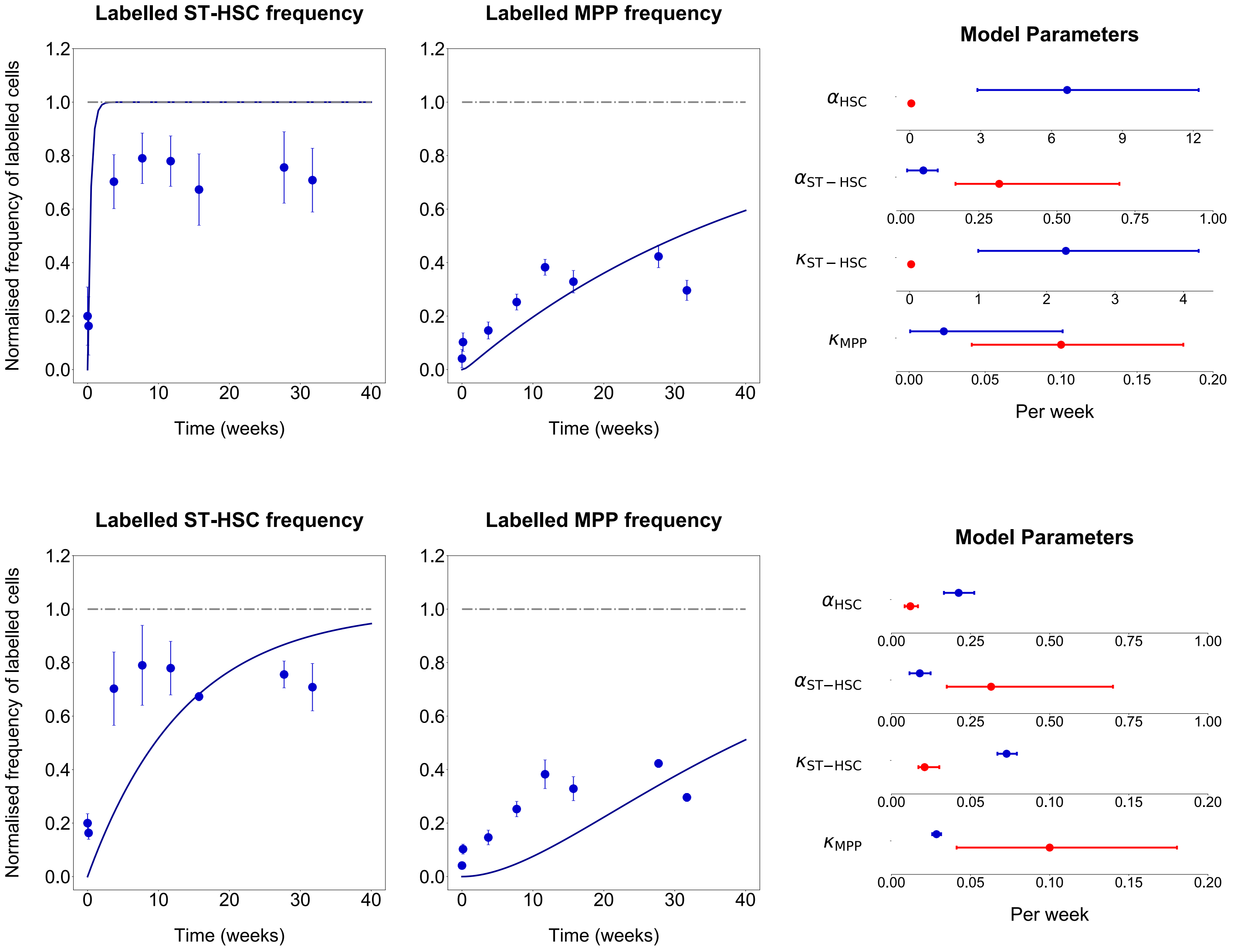

B

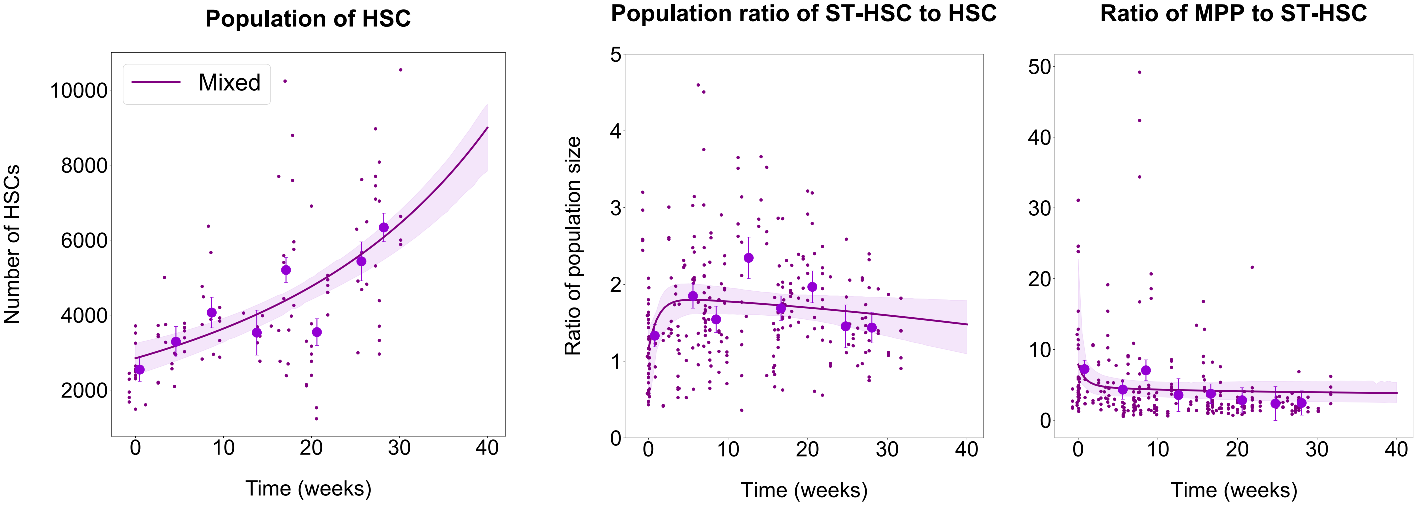

Figure S1

A) Label propagation results cannot be fit to previous models. Refer to Figure 1C;
Initial model fit when applying Busch model to *Fgd5* dataset (big dots representing average and SEM of datasets, small dots show mice data from independent experiments n=48). Top panel: Using standard error, model overfits to single point of dataset. Bottom panel: With pooled variance, model is not able to fit the data well, with large error bounds in calculated parameters.

B) Model is able to fit stem cell population dynamics. Refer to Figure 2B;
Model best fit (solid line) and 95% prediction profile likelihood confidence bounds on the model fit on *Fgd5* and *Tie2* dataset (shaded area) plotted against the experimental data (big dots representing average and SEM of two datasets, small dots show mice data from independent experiments n=113 for first plot and n=291 for remaining two plots).

Supplemental Figure 2

A

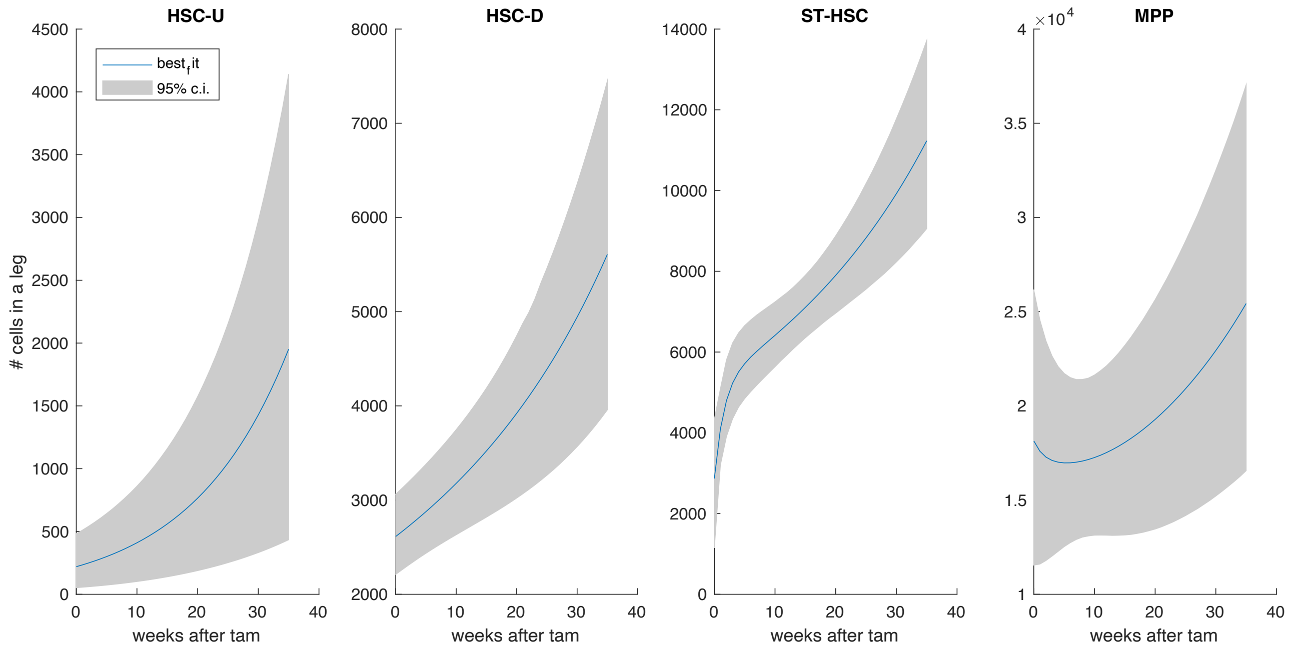

Figure S2

A) Model predicted stem cell dynamics. Refer to Figure 2B;

Model best fit (solid line) and 95% prediction profile likelihood confidence bounds on the model fit for stem cell populations.

Supplemental Figure 3

A ***Fgd5* labelled mice**

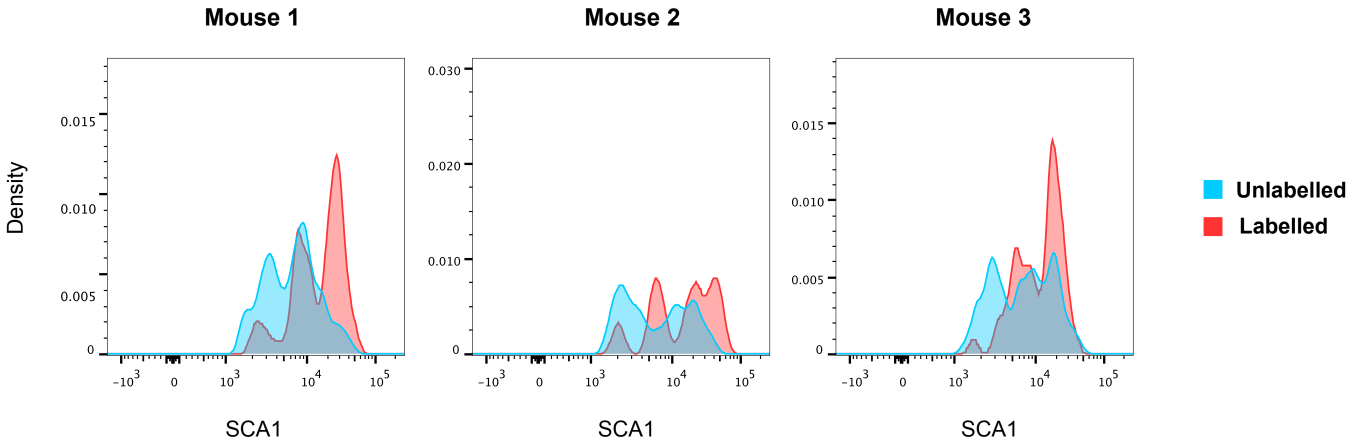

***Krt18* labelled mice**

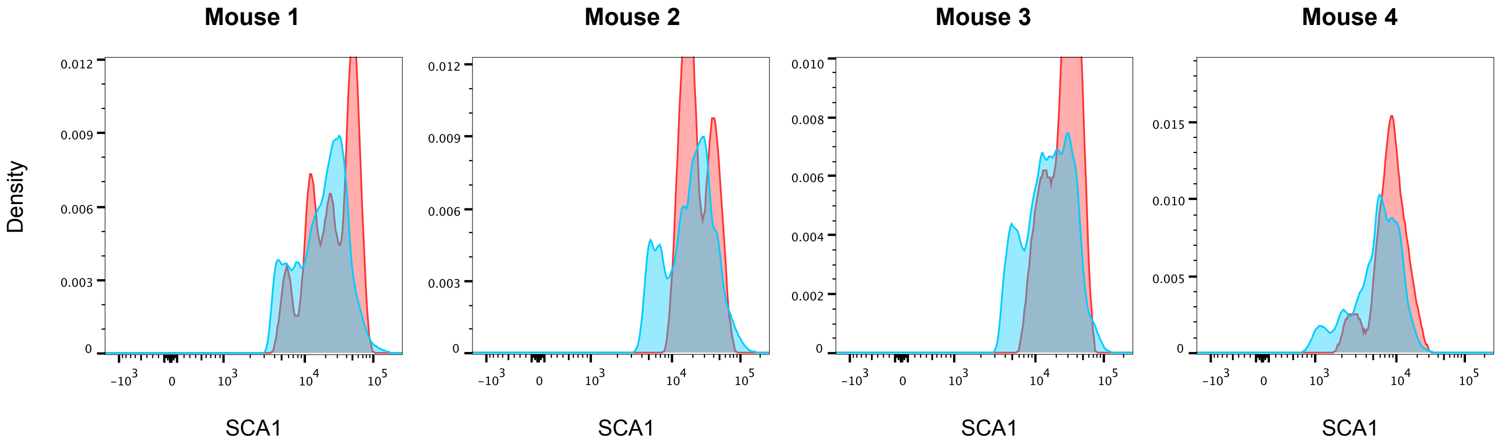

Figure S3

A) SCA1 expression density plots for HSCs in *Fgd5* and *Krt18* fate mapping experiments for all mice. Refer to Figure 3C;

Plot shows comparison of the distribution between labelled and unlabelled HSC cells for each reporter gene at first measured time point after induction (7 days) for all mice. Data measured at same lab on different days.

Supplemental Methods: Model Fit

The model equations were fitted to experimental data by Busch et al., 2015 and Säwén et al., 2018:

- For the label frequency, data points from Busch et al., 2015 (over 100 mice) and Säwén et al., 2018 (50 mice) were considered: $f^{\mathrm{o}}_{\mathrm{Tie2},i}(t_j)$ and $f^{\mathrm{o}}_{\mathrm{Fgd5}\,,i}(t_k)$ respectively and similarly, their pooled variance was considered: $\delta f^{\mathrm{o}}_{\mathrm{Tie2},i}(t_j)$ and $\delta f^{\mathrm{o}}_{\mathrm{Fgd5}\,,i}(t_k)$, where j and $k = 1{:}\,8$.
- For the size of populations, the combined population data from both datasets (over 100 mice) along with their pooled variance were considered to calculate the ratios: $r_i^{\mathrm{o}}(t_m)$ and $\delta r_i^{\mathrm{o}}(t_m)$,where $r_i^{\mathrm{o}}(t_m) = \; n_i^{\mathrm{o}}(t_m)/n_{i-1}^{\mathrm{o}}(t_m)$ and $m = 1{:}\,8$. However, since the ratios did not reflect the heterogeneity of the HSC population, $n^{\mathrm{o}}_{\mathrm{HSC}}(t_m)$ and $\delta n^{\mathrm{o}}_{\mathrm{HSC}}(t_m)$ were considered separately.

The best set of parameters was found via the *scipy.optimize.least_squares* Python tool. It runs on the Levenberg-Marquardt and trust-region-reflective algorithms to find a local minimum for the cost function

$$
\begin{aligned}
&c\left(\alpha, \kappa, f_{\mathrm{Tie2},1-4}(0), f_{\mathrm{Fgd5},1-4}(0), n(0)\right) \\
&\quad = \sum_{m=1}^{8}\left(\frac{(n_0(t_m) + n_1(t_m)) - n^{\mathrm{o}}_{\mathrm{HSC}}(t_m)}{\delta n^{\mathrm{o}}_{\mathrm{HSC}}(t_m)}\right)^2 \\
&\quad + \sum_{i=3}^{4}\sum_{m=1}^{8}\left(\frac{n_i(t_m) - n_i^{\mathrm{o}}(t_m)}{\delta n_i^{\mathrm{o}}(t_m)}\right)^2 + \sum_{i=1}^{4}\sum_{j=1}^{8}\left(\frac{f_{\mathrm{Tie2},i}(t_j) - f^{\mathrm{o}}_{\mathrm{Tie2},i}(t_j)}{\delta f^{\mathrm{o}}_{\mathrm{Tie2},i}(t_j)}\right)^2 \\
&\quad + \sum_{i=1}^{4}\sum_{k=1}^{8}\left(\frac{f_{\mathrm{Fgd5},i}(t_k) - f^{\mathrm{o}}_{\mathrm{Fgd5},i}(t_k)}{\delta f^{\mathrm{o}}_{\mathrm{Fgd5},i}(t_k)}\right)^2 \qquad (6)
\end{aligned}
$$

Finding a global minimum was guaranteed by starting the optimization from randomly generated initial guesses for the parameters in the space sample. A self-implemented profile

likelihood framework based on Raue et al., 2009 was implemented to estimate the 95% confidence bounds on the parameters. The 95% confidence bounds on the model were similarly found via the prediction profile likelihood framework based on Kreutz et al., 2012.

To test the model on an unseen dataset, further experimental data from Chapple et al., 2018 (18 mice) was used to produce a fit:

- For the label frequency and pooled variance, $f^{\mathrm{o}}_{\mathrm{Krt18},i}(t_n)$ and $\delta f^{\mathrm{o}}_{\mathrm{Krt18},i}(t_n)$ were considered where $n = 4$.

The cell kinetic parameters (i.e. α, κ) and the initial condition of the populations' size (i.e. $n(0)$) were fixed. The cost function was minimized, and model plotted to test fit.

$$c\left(f_{\mathrm{Krt18},1-4}(0)\right) = \sum_{i=1}^{4}\sum_{j=1}^{4}\left(\frac{f_{\mathrm{Krt18},i}(t_j) - f^{\mathrm{o}}_{\mathrm{Krt18},i}(t_j)}{\delta f^{\mathrm{o}}_{\mathrm{Krt18},i}(t_j)}\right)^2 \qquad (7)$$

Parameters were bounded to ensure that biologically meaningful estimation were obtained. (i.e. initial frequencies were constrained to be a value between 0 and 1, population size to be positive, and, on the basis that a cell cycle is not faster than 6 hours, α values to be positive and less than 28 per week, κ values to be between -28 and 56).

Since Barile et al. 2020 observed that α_{ST} must be greater than κ_{ST}, this constraint was added as a further boundary condition to compute the parameter estimates.
